# Chitosan Oligosaccharides Improve Glucolipid Metabolism Disorder in Liver by Suppression of Obesity-Related Inflammation and Restoration of Peroxisome Proliferator-Activated Receptor Gamma (PPARγ)

**DOI:** 10.3390/md16110455

**Published:** 2018-11-19

**Authors:** Yibo Bai, Junping Zheng, Xubing Yuan, Siming Jiao, Cui Feng, Yuguang Du, Hongtao Liu, Lanyan Zheng

**Affiliations:** 1 Department of Pathogen Biology, College of Basic Medical Sciences, China Medical University, No. 77 Puhe Road, Shenyang North New Area, Shenyang 110122, China; YiboBaicmu@163.com; 2 State Key Laboratory of Biochemical Engineering and Key Laboratory of Biopharmaceutical Production & Formulation Engineering, PLA, Institute of Process Engineering, Chinese Academy of Sciences, Beijing 100190, China; junpingzheng2013@163.com (J.Z.); xbyuan@ipe.ac.cn (X.Y.); smjiao@ipe.ac.cn (S.J.); cfeng@ipe.ac.cn (C.F.); 3 Zhengzhou Institute of Emerging Industrial Technology, Zhengzhou 450000, China

**Keywords:** chitosan oligosaccharide, glucolipid metabolism disorder, high-fat diet, inflammation, peroxisome proliferator-activated receptor gamma

## Abstract

Chitosan oligosaccharides (COS) display various biological activities. In this study, we aimed to explore the preventive effects of COS on glucolipid metabolism disorder using palmitic acid (PA)-induced HepG2 cells and high-fat diet (HFD)-fed C57BL/6J mice as experimental models in vitro and in vivo, respectively. The results showed that COS pretreatment for 12 h significantly ameliorated lipid accumulation in HepG2 cells exposed to PA for 24 h, accompanied by a reversing of the upregulated mRNA expression of proinflammatory cytokines (IL-6, MCP-1, TNF-α) and glucolipid metabolism-related regulators (SCD-1, ACC1, PCK1-α). In addition, COS treatment alleviated glucolipid metabolism disorder in mice fed with HFD for five months, including reduction in body weight and fasting glucose, restoration of intraperitoneal glucose tolerance, and suppression of overexpression of proinflammatory cytokines and glucolipid metabolism-related regulators. Furthermore, our study found that COS pretreatment significantly reversed the downregulation of PPARγ at transcriptional and translational levels in both PA-induced HepG2 cells and liver tissues of HFD-fed mice. In summary, the study suggests that COS can improve glucolipid metabolism disorder by suppressing inflammation and upregulating PPARγ expression. This indicates a novel application of COS in preventing and treating glucolipid metabolism-related diseases.

## 1. Introduction

Data shows that over 30% of adults and 16.9% of children in the US are obese or overweight, and the figures are 14.3% and 12.8% in China, respectively [1,2]. Obesity is usually accompanied by numerous complications, such as metabolic syndrome, nonalcoholic fatty liver diseases (NAFLDs), cardiocerebrovascular diseases, and cancers [3]. At present, lifestyle intervention, drug treatment, and surgical operation are the main therapies used for obesity, but the safety and effectiveness of these methods are unsatisfactory [4,5]. Thus, an efficient preventive strategy is necessary to improve glucolipid metabolism disorder without being confined to search for effective treatments.

Chitosan oligosaccharides (COS) are a kind of multifunctional oligosaccharides. COS are produced from chitosan, a polysaccharide constituent of crustaceans such as shrimps, crabs, lobsters, and prawns, by enzymatic degradation or acidic hydrolysis [6]. COS are known to display various bioactivities, such as anti-inflammation, anticancer, and antioxidation [7,8,9]. In addition, studies have shown that both chitosan and COS could improve overweight and dyslipidemia in rats [10,11,12]. Regretfully, the preventive effect of COS on metabolism disorder and the underlying molecular mechanism have failed to be fully elucidated. Recently, chitosan was reported to decrease the number of *Firmicutes* and *Lactobacillus* spp. in the caecum and colon but increase the population of *Bifidobacteria* in caecum of pigs [13], indicating that chitosan might be a kind of prebiotics. Unlike chitosan, COS are totally deacetylated polymers of *N*-acetylglucosamine, which have shorter chain length and lower molecular weights [6]. Compared to chitosan, COS have been shown to have higher solubility, lower viscosity, and higher absorption rate in both in vitro and in vivo transport experiments [14]. Based on the above, COS should be easily transported across the gastrointestinal tract and absorbed into the blood flow, where they display biologic effects on metabolic diseases.

So far, several functional polysaccharides have been proven to reverse glucose and lipid metabolism disorders by upregulating the activity of nuclear receptor peroxisome proliferator-activated receptor gamma (PPARγ) [15,16,17]. PPARγ is an important member of the nuclear receptor super family of transcription factors, standing at the crossroads of controlling metabolic disorders, including obesity, insulin resistance, and cardiovascular diseases [18]. PPARγ was first found to be responsible for adipocyte differentiation [19]. In recent years, it has been reported that PPARγ can improve lipid metabolism and insulin sensitivity through regulation of inflammatory mediators and plentiful enzymes involved in lipid synthesis, uptake, and release [20,21]. Studies have also shown that activation of PPARγ alleviates liver injury and liver fibrosis [22,23]. PPARγ was also found to prevent the progression of hepatic steatosis in mouse models [24,25]. Therefore, PPARγ has been regarded as a drug target against metabolic diseases [18,26].

The purpose of this study was to determine whether COS administrated in advance could alleviate obesity-associated liver lipid metabolic disorder, both in vitro and in vivo, and if so, to determine the mechanism involved. Because of the critical role of PPARγ in the regulation of lipid metabolism, we specifically examined the effect of COS on PPARγ in palmitic acid (PA)-induced HepG2 cells and high-fat diet (HFD)-induced mice.

## 2. Result

### 2.1. Effect of COS and PA Treatment on HepG2 Cells Viability

**Figure 1:**
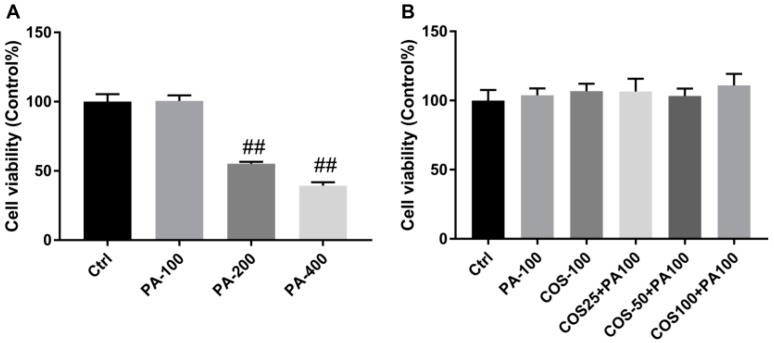
Effect of palmitic acid (PA) and chitosan oligosaccharides (COS) on viability of HepG2 cells. (**A**) HepG2 cells were treated with PA (100–400 µM) for 24 h. (**B**) HepG2 cells were treated with COS (100 µg/mL) for 12 h or pretreated with COS (25–100 µg/mL) for 12 h and then exposed to PA (100 µM) for 24 h. After that, the cell viability was determined by MTT assay. The data presented are averages and standard deviations of three independent experiments, quintuplicate in each experiment. ## *p* < 0.01, compared to the control group.

We first evaluated the effect of COS and PA on HepG2 cells viability by the 3-(4,5-dimethylthiazol-2-yl)-2,5-diphenyltetrazolium bromide (MTT) assay. As shown in Figure 1A, PA at 100 μM had no effect on HepG2 cells viability after treatment for 24 h, while higher concentrations of PA (200,400 μM) resulted in significant decrease in the viability of HepG2 cells (*p* < 0.01, vs. the control group). On the other hand, the incubation of COS (100 μg/mL) for 12 h or pretreatment with 25, 50, and 100 μg/mL COS for 12 h and then exposure to 100 μM of PA for 24 h both caused no toxicity to HepG2 cells (Figure 1B). Therefore, 100 μM of PA and 25–100 μg/mL of COS were chosen for further experiments.

### 2.2. COS Ameliorated PA-Induced Lipid Accumulation in HepG2 Cells

**Figure 2:**
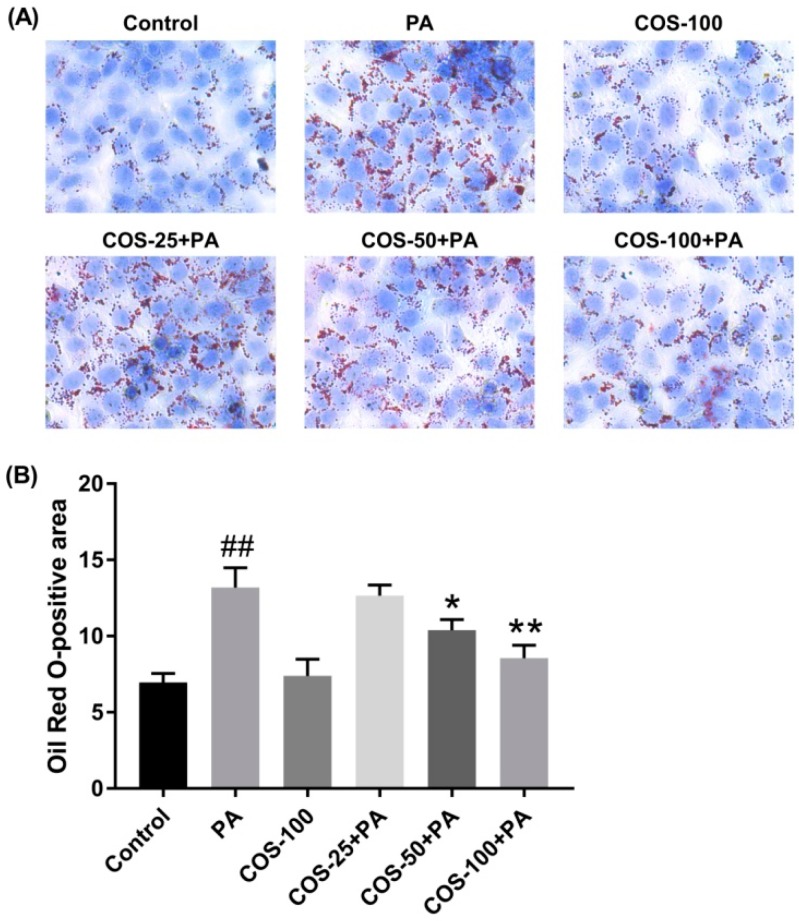
Effects of COS on PA-induced lipid deposition in HepG2 cells. (**A**) Cells were pretreated with COS (25–100 μg/mL) for 12 h and then exposed to PA (100 μM) for 24 h. Finally, cells were stained with oil red O, and the visualized red oil droplets were observed using a Leica light microscope. The photographs are representative of three independent experiments with similar results, triplicate in each experiment. (**B**) Quantitative data for the percentage of lipid droplets in HepG2 cells (oil red O-positive areas) shown as a histogram. The data presented are averages and standard deviations of three independent experiments. ## *p* < 0.01, compared to the control group; * *p* < 0.05 or ** *p* < 0.01 compared to the PA group.

To investigate whether COS could alleviate PA-induced lipid accumulation, HepG2 cells were pretreated with COS (25–100 μg/mL) for 12 h and then exposed to PA (100 μM) for 24 h. The results by oil red O staining showed that PA induced a considerable lipid accumulation in HepG2 cells, which was suppressed by COS pretreatment in a concentration-dependent manner (Figure 2).

### 2.3. COS Reversed the upregulation of Proinflammatory Cytokines and Glucolipid Metabolism-Related Regulators at mRNA Level in PA-Induced HepG2 Cells

Considering that obesity is associated with low-grade chronic inflammation [3], we next detected the transcriptional levels of inflammatory cytokines in HepG2 cells with PA or PA plus COS treatment by RT-PCR.

**Figure 3:**
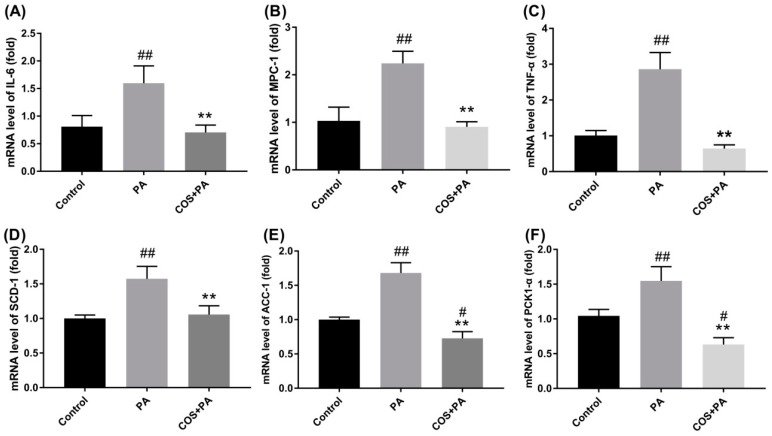
Effects of COS on PA-induced proinflammatory cytokines and fatty acid metabolism-related regulators in HepG2 cells. Cells were pretreated with COS (100 μg/mL) for 12 h and then exposed to PA (100 μM) for 24 h. After that, the mRNA levels of (**A**) IL-6, (**B**) MCP-1, (**C**) TNF-α, (**D**) SCD-1, (**E**) ACC-1, and (**F**) PKC1-α were determined by RT-PCR. The data presented are averages and standard deviations of three independent experiments with similar results, triplicate in each experiment. # *p* < 0.05 or ## *p* < 0.01, compared to the control group; ** *p* < 0.01 compared to the PA group.

As shown in Figure 3A–C, PA (100 μM) treatment for 24 h obviously activated the mRNA expression of IL-6, MCP-1, and TNF-α (*p* < 0.01, vs. the control group) in HepG2 cells. In contrast, the PA-induced inflammation was remarkably suppressed by COS (100 μg/mL) pretreatment for 12 h (*p* < 0.01, vs. the PA group).

On the other hand, PA treatment (100 μM) for 24 h significantly increased the mRNA levels of fatty acid synthesis-related regulators (stearoyl-CoA desaturase, SCD-1, and acetyl-CoA carboxylase, ACC1) and glucogenesis-associated regulator PCK1-α (*p* < 0.01, vs. the control group) in HepG2 cells, which were evidently inhibited by COS (100 μg/mL) pretreatment for 12 h (Figure 3D–F).

When these factors in PA plus COS treatment group were compared with the control group, no significant differences were observed (Figure 3A–D) except for the ACC-1 (Figure 3E) and PCK1-α (Figure 3F). To exclude unexpected accidents, we repeated these experiments more than three times independently and got consistent results. A review of the literature shows that analogous results were generated by other groups [12,27]. For example, in one study, COS treatment initiated less expression of proinflammatory cytokines in LPS-treated endothelial cells compared to the control group [27]. Another study found COS stimulated stronger leptin signaling transduction in obese rats [12]. Unfortunately, there is no plausible explanation about the mechanism involved thus far. Although this matter is out of the scope of this study, it deserves further investigation in the future.

### 2.4. COS Reversed the downregulation of PPARγ at Both mRNA and Protein Levels in PA-Induced HepG2 Cells

**Figure 4:**
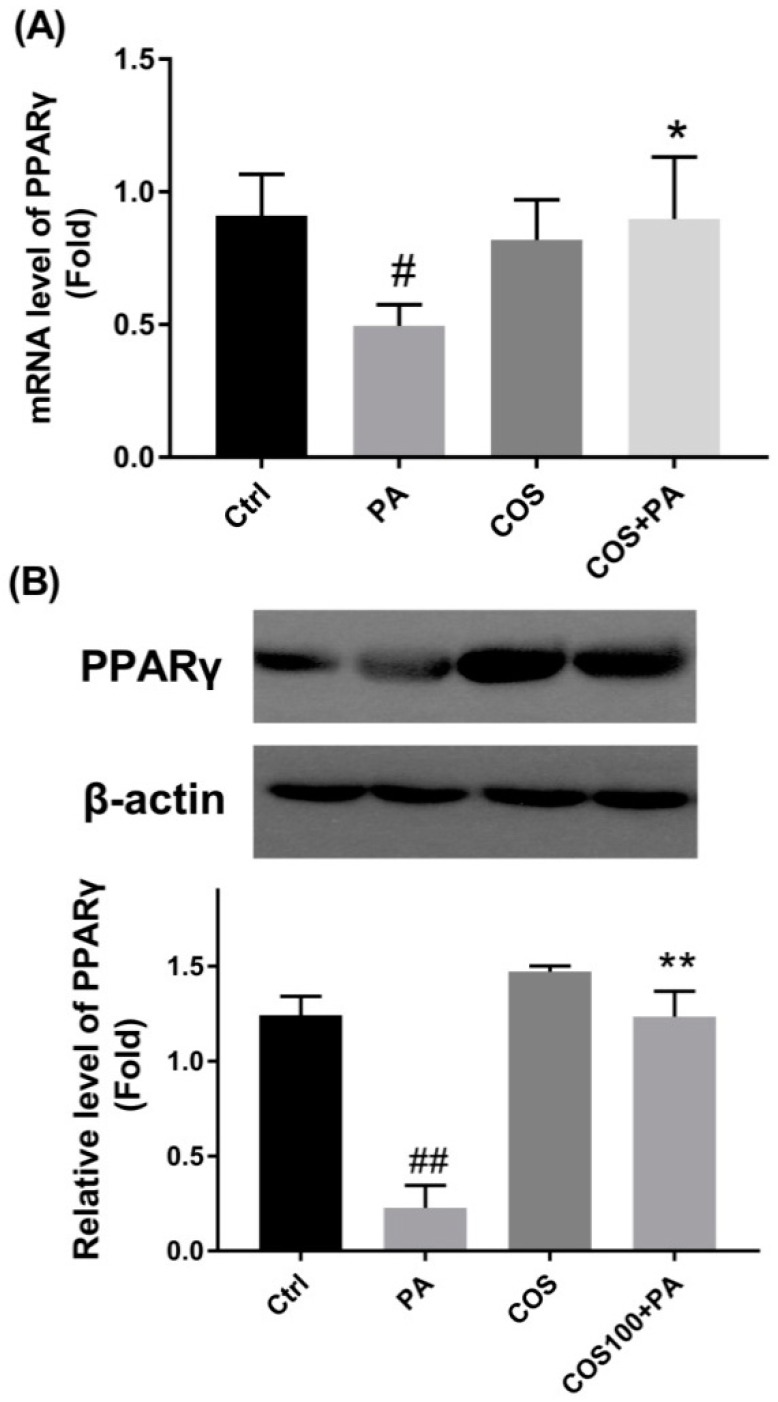
Effects of COS on PA-induced downregulation of PPARγ in HepG2 cells. Cells were pretreated with COS (100 μg/mL) for 12 h and then exposed to PA (100 μM) for 24 h. (**A**) The mRNA level of PPARγ as determined by RT-PCR. (**B**) The protein level of PPARγ as measured by western blot (WB) analysis. The data presented are representative images of three independent experiments with similar results. Data are represented as the mean ± SD (*n* = 3). # *p* < 0.05 or ## *p* < 0.01, compared to the control group; * *p* < 0.05 or ** *p* < 0.01 compared to the PA group.

To explore the potential molecular mechanism by which COS prevent PA-induced inflammation and glucolipid metabolism disorder, we measured the expression changes of PPARγ in PA-induced HepG2 cells, which plays a key role in the regulation of lipid metabolism. The results in Figure 4 shows that PA (100 μM) exposure for 24 h led to a distinct reduction in PPARγ at both mRNA and protein levels in HepG2 cells (*p* < 0.05 or 0.01, vs. the control group). However, after COS pretreatment (100 μg/mL) for 12 h, the decreased expressions of PPARγ were statistically reversed in PA-induced HepG2 cells.

### 2.5. COS Alleviated Glucose Intolerance in HFD-Fed Mice

**Figure 5:**
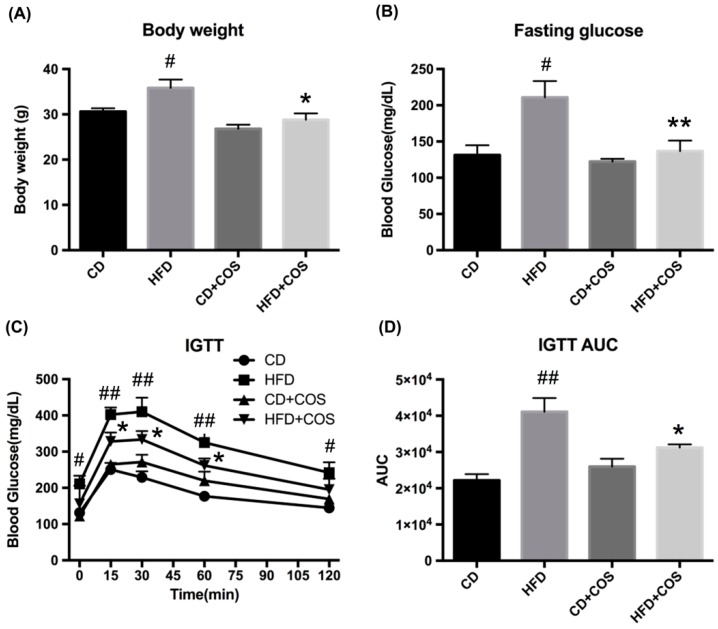
Effects of COS on body weight, fasting glucose, intraperitoneal glucose tolerance test (IGTT), and area under curve (AUC) of IGTT in HFD-fed mice. C57BL/6J mice were fed with control diet (CD), high-fat diet (HFD), CD plus COS (1 mg/mL in drinking water), or HFD plus COS for five months. After the treatment, the (**A**) body weight and (**B**) fasting glucose were monitored. (**C**) The IGTT was also measured, and (**D**) the AUC of IGTT was calculated. Data are represented as the mean ± SD (*n* = 5). # *p* < 0.05 or ## *p* < 0.01, compared to the CD group; * *p* < 0.05 or ** *p* < 0.01 compared to the HFD group.

To study the effects of COS on glucolipid metabolism disorder in vivo, C57BL/6J mice were fed with control diet (CD), HFD, CD plus COS (1 mg/mL in drinking water), or HFD plus COS for five months. The results showed that COS significantly lowered HFD-induced increase in body weight (*p* < 0.05, vs. the HFD group) (Figure 5A) and fasting glucose level (*p* < 0.01, vs. the HFD group) (Figure 5B). In addition, intraperitoneal glucose tolerance test (IGTT) suggested that the mice in HFD group displayed poorer behavior in terms of glucose tolerance compared to the CD group, while COS treatment remarkably alleviated glucose intolerance in HFD-fed mice (Figure 5C). Furthermore, the area under curve (AUC) of glucose in the IGTT was found to be much higher in the HFD group than the control group (*p* < 0.01), while COS treatment obviously reduced the AUC in HFD-fed mice, which was almost comparable to the CD group (Figure 5D).

### 2.6. COS Treatment Ameliorated Glucolipid Metabolism Disorder in HFD-Fed Mice

**Figure 6:**
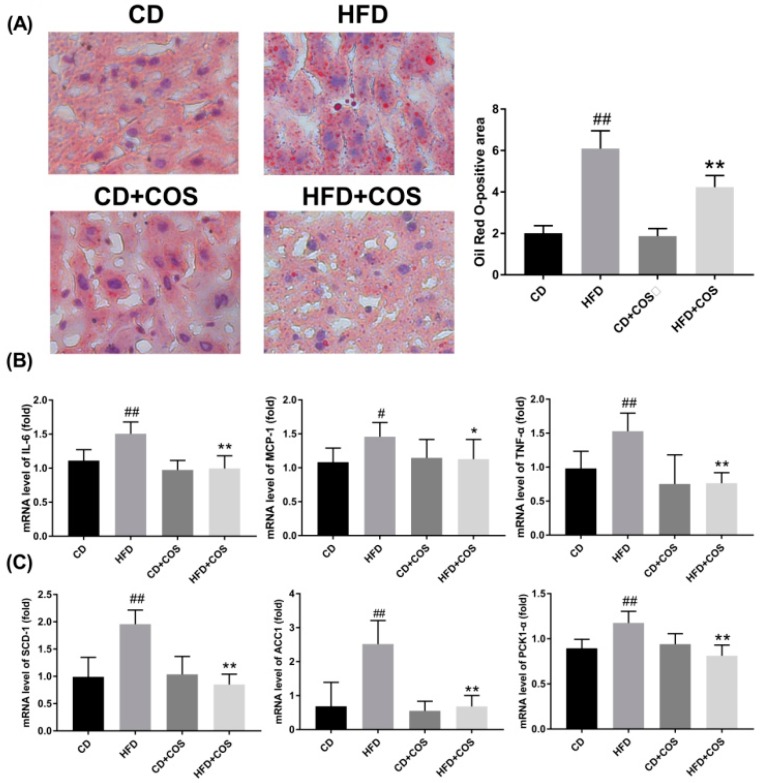
Improvement in lipid metabolism disorder by COS in HFD-fed mice. C57BL/6J mice were fed with CD, HFD, CD plus COS (1 mg/mL in drinking water), or HFD plus COS for five months. After the treatment, all mice were sacrificed and the liver tissues were collected. Next, the lipid deposits in hepatocytes were observed by oil red O staining (**A**). Quantitative data for the percentage of oil deposits in the liver (oil red O-positive areas) as calculated by Image j. (**B**) The mRNA levels of proinflammatory cytokines (IL-6, MCP-1, TNF-α) and (**C**) fatty acid metabolism-related regulators (SCD-1, ACC-1, PKC1-α) in liver as determined by RT-PCR. Data are represented as the mean ± SD (*n* = 5). # *p* < 0.05 or ## *p* < 0.01, compared to the CD group; * *p* < 0.05 or ** *p* < 0.01 compared to the HFD group.

To explore the effect of COS on hepatic steatosis, the lipid accumulation in all groups was measured using oil red O staining. As indicated in Figure 6A, a mass of lipid droplets were observed after staining in the hepatocytes of liver tissues of HFD-fed mice, and the lipid accumulation was hampered in HFD-fed mice with COS treatment.

Next, the effect of COS on inflammatory responses in HFD-fed mice was assessed. As shown in Figure 6B, HFD feeding caused significant increase in mRNA expression of three proinflammatory cytokines (IL-6, MCP-1, and TNF-α) in liver tissues (*p* < 0.05 or 0.01, vs. the CD group), which was reversed by COS treatment (*p* < 0.05 or 0.01, vs. the HFD group). Likewise, COS treatment led to evident transcriptional inhibition of glucolipid metabolism-related regulators (SCD-1, ACC1, and PCK1-α) in liver tissues of HFD-fed mice (*p* < 0.01) (Figure 6C).

**Figure 7:**
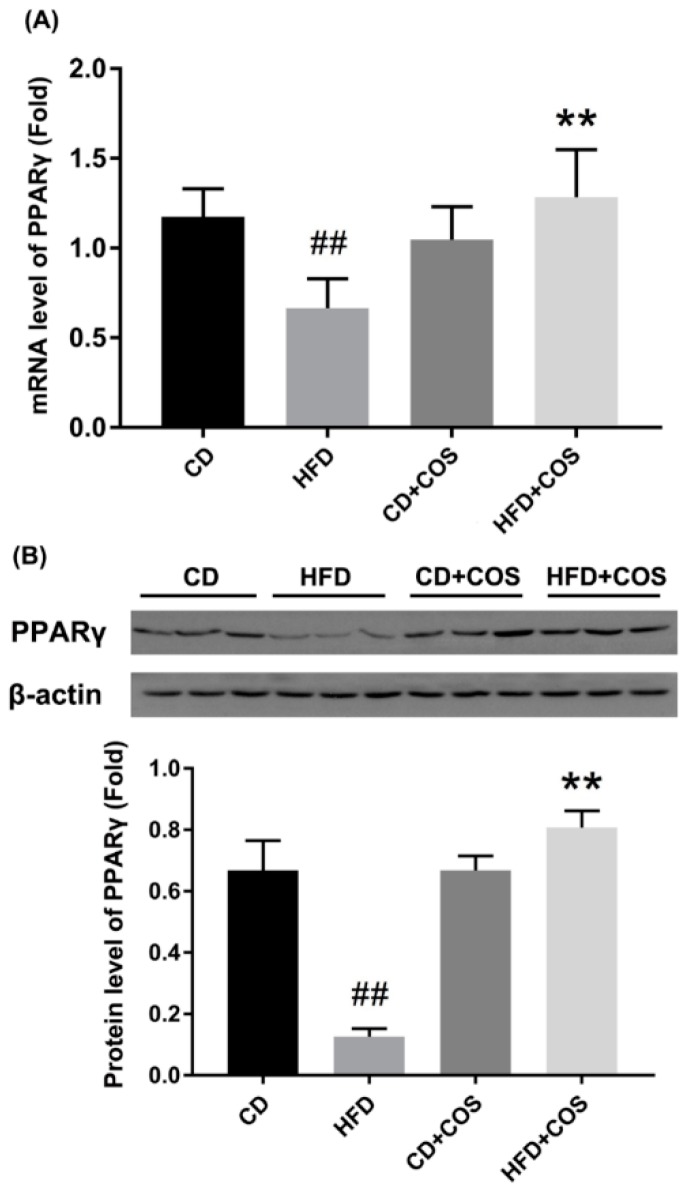
Reversal of downregulated PPARγ by COS in liver tissues of HFD-fed mice. C57BL/6J mice were fed with CD, HFD, CD plus COS (1 mg/mL in drinking water), or HFD plus COS for five months. After the treatment, all mice were sacrificed and the liver tissues were collected. The expressions of PPARγ (**A**) at transcriptional level as determined by RT-PCR, and (**B**) at translational level as determined by WB analysis. Data are represented as the mean ± SD (*n* = 5). ## *p* < 0.01, compared to the CD group; ** *p* < 0.01 compared to the HFD group.

Finally, to further confirm the role of PPARγ in COS-mediated improvement of glucolipid metabolism disorder in HFD-fed mice, we measured the variation of PPARγ in liver tissues of all experimental groups. As shown in Figure 7, both the transcription and the translation of PPARγ were significantly downregulated in liver tissues of HFD-fed mice compared to the CD group (*p* < 0.01). However, COS treatment obviously reversed the reduction of PPARγ expression (Figure 7).

## 3. Discussion

Obesity associated with NAFLD, diabetes, and cancer has become a major global health challenge [3]. Currently, the main therapies against obesity include lifestyle intervention, drug treatment, and surgical operation [4,5]. However, the efficacy and safety of these methods are of concern. Thus, it is urgently necessary to identify alternative therapeutic methods to curb the increasing incidence of obesity. Recently, a series of polysaccharides or oligosaccharides as prebiotics were found to alleviate glucolipid metabolism disorders, and good results were obtained [13,28,29]. Among them, COS were reported to display excellent antioxidative and anti-inflammatory effects [7,30]. Additionally, COS were shown to have antiobese effect in HFD-feed rats [10,11,12]. However, the preventive effect of COS on metabolism disorder and the molecular mechanism remain obscure. As COS can be easily absorbed into the blood flow [14], we speculated that COS might directly initiate the regulatory effect on glucolipid metabolism. In this study, we found that COS improved HFD-induced glucose intolerance and dyslipidemia by restoring the downregulated PPARγ in PA-induced HepG2 cells or HFD-fed mice.

In this study, we used PA-induced HepG2 cells as in vitro model to investigate the preventive function of COS on lipid stress. Besides the biosynthetic capacity of plasma proteins and inflammatory mediators, HepG2 cells can express most of the cellular surface receptors of normal hepatocytes and are more stable than the latter in vitro [31]. Therefore, HepG2 cells are usually chosen to study liver-related metabolism diseases instead of primary hepatocytes. In addition, as the most abundant nonesterified fatty acids in plasma, PA is the most widely used inducer in the investigation of lipid metabolism [32]. In line with the published results [32,33], our studies showed that PA not only led to lipid droplet deposition and activation of the inflammatory response, but it also dramatically affected the expression of glucolipid metabolism-related regulators in HepG2 cells.

Liver plays an important role in lipid metabolism by which excessive dietary lipids are stored in hepatocytes [34]. This further impairs the metabolism of glucose and fatty acids, even leading to the occurrence of insulin resistance. In the present study, both HepG2 cells exposed to PA and mice chronically subjected to HFD exhibited evident lipid droplet accumulation, suggesting the formation of dyslipidemia (Figure 2 and Figure 6A). On the contrary, the imbalance of lipid metabolism in both HepG2 cells and liver tissues of HFD-fed mice was reversed by COS treatment (Figure 2 and Figure 6A). In particular, COS displayed significantly inhibitory effect on glucose intolerance in mice with HFD feeding (Figure 5C,D). These results are parallel to our previous study in which chitin oligosaccharides, the acetylated form of COS, was proven to ameliorate HFD-induced dyslipidemia in mice [35]. Based on the above, it is suggested that COS could attenuate the metabolism syndrome associated with obesity.

Glucolipid metabolism disorder related to systemically upregulated chronic inflammatory responses and obesity is also characterized by increasing systemic inflammation and insulin resistance [36]. Thus, attempts have been made to repress metabolic diseases through the blockade of inflammatory responses. It has been reported that PA overload activates inflammatory signaling to produce cytokines, such as IL-6, MCP-1, and TNF-α [37,38]. Our results showed that COS significantly downregulated the overexpression of IL-6, MCP-1, and TNF-α in PA-induced HepG2 cells as well as in liver tissues of HFD-fed mice at the mRNA level (Figure 3A–C and Figure 6B). Considering that the increased influx of hepatic free fatty acids impairs insulin signaling, stimulates hepatic gluconeogenesis, and activates the de novo lipogenesis [39], we next investigated whether COS treatment could affect the expression of glucolipid metabolism-related regulators, i.e., PCK1-α, SCD-1, and ACC-1. PCK1-α is one of the key gluconeogenic enzymes, while SCD-1 is a microsomal enzyme required for the synthesis of oleate and palmitoleate, and ACC-1 is a major enzyme in de novo fatty acid biosynthetic pathway [40,41,42]. Our results indicated that overexpression of the three regulators was drastically inhibited by COS treatment at the mRNA level both in vitro and in vivo (Figure 3D–F and Figure 6C), indicating the suppressive effect of COS on gluconeogenesis and free fatty acid synthesis in hepatocytes and liver tissues with overflowing fatty acids. Based on the above, we propose that COS improve glucolipid disorder in obesity mice, perhaps by suppressing inflammation.

PPARγ is an important transcription factor responsible for lipid metabolism and inflammatory responses [18], which has been proven to regulate the expression of various metabolic enzymes involved in lipid synthesis and fatty acid β-oxidation [20]. A previous study demonstrated that hepatic PPARγ mRNA and protein expression level decreased in NAFLD rats compared to the controls [43]. In livers of diabetic nephropathy rat and db/db mice, the protein level of PPARγ was shown to decrease, and restoring PPARγ gene expression to baseline could improve metabolic disorders [15,44]. Therefore, a balanced level of PPARγ in liver tissues may be important for homeostasis. In addition, PPARγ activation has been reported to inhibit the expression of inflammatory mediators by blocking the NF-κB [45,46,47]. Here, we explored whether PPARγ was critical to COS-mediated improvement of glucolipid metabolic disorder. Our study showed that PA induced a significant decrease in the expression of PPARγ at both mRNA and protein levels in HepG2 cells, which was almost totally reversed by COS treatment (Figure 4). Similar results were identified in the liver tissues of HFD-fed mice (Figure 7). These results are in line with findings from other groups, which demonstrated that some polysaccharides or their derivatives prevented the occurrence of metabolic diseases by upregulating the expression of PPARγ [15,16,17]. From the above, we speculate that PPARγ may be a potential molecular target, with COS initiating the protective effect on glucolipid metabolism disorder. In conclusion, we proved that COS displayed strong improvement on glucolipid metabolism disorder in PA-induced HepG2 cells as well as liver tissues of HFD-fed mice. This molecular mechanism might be associated with the reversal effect of COS on reduced PPARγ production, which subsequently downregulated the overexpression of proinflammatory cytokines and inhibited the activation of gluconeogenesis and lipogenesis in hepatocytes with overflowing fatty acids. This study suggests a novel application of COS to prevent and treat glucolipid metabolism-related diseases.

## 4. Materials and Methods

### 4.1. Chemicals and Reagents

HepG2 cells were supplied by the Chinese Academy of Sciences Cell Bank (Shanghai, China). COS were prepared in our laboratory [48]. PA was purchased from Thermo Fisher Scientific (Waltham, MA, USA). Minimum essential medium (MEM), penicillin/streptomycin, and nonessential amino acids (NEAA) were obtained from Gibco (Grand Island, NY, USA). Fetal bovine serum (FBS) was purchased from Kang Yuan biology company (Beijing, China). Anti-PPARγ, anti-β-actin, horseradish peroxidase (HRP)-conjugated goat anti-rabbit IgG, and HRP-conjugated goat anti-mouse IgG were obtained from Cell Signaling Biotechnology (Beverly, MA, USA).

### 4.2. Cell Culture and Drug Treatment

HepG2 cells were maintained in MEM containing 10% FBS and 1% NEAA, 1% penicillin–streptomycin at 37 °C in humidified atmosphere with 5% CO_2_. After reaching subconfluence, cells were incubated with 0, 25, 50, 100 μg/mL COS for 12 h and then exposed to 100 μM of PA diluted in culture medium for 24 h at 37 °C for further assay.

### 4.3. Cell Viability Assay

HepG2 cells were plated at density of 1 × 10^4^ cells/well in 96-well plates for 24 h. After treatment with PA at concentrations of 100 μM, 200 μM, and 400 μM or COS (100 μg/mL) for 24 h, the toxic effects of PA or COS alone on cell viability were determined. To assess the effect of COS plus PA on cell viability, the HepG2 cells were separately pretreated with 25, 50, and 100 μg/mL COS for 12 h and then exposed to 100 μM of PA for 24 h. After that, the cells were washed with PBS and incubated with MTT (5 mg/mL) in culture medium for 3 h at 37 °C. Next, the medium was discarded, and the formazan blue, which formed inside the cells, was dissolved using 100 μL dimethyl sulfoxide (DMSO). The optical density at 490 nm was determined with a Sunrise Remote Microplate Reader (Grodig, Austria). The experiments were repeated 3 times independently, and 5 replicates were involved in each sample. The cell viability of each well was presented as the percentage of control level.

### 4.4. Animal Experiment

C57BL/6J wild-type male mice were obtained from Vital River Laboratory Animal Technology Co., Ltd. (Beijing China) and kept in a room with a 12 h light/dark cycle, a temperature of 22 ± 2 °C, and a relative humidity of 50 ± 5% during the whole experiment period. Mice were fed with a control diet for two weeks for adaptation. Then, all mice were randomly divided into four groups (*n* = 5): CD group, HFD group, CD + COS (1 mg/mL in drinking water, about 200 mg/kg/d) group, and HFD + COS group. The HFD was composed of 60% basic feed, 10% lard, 10% egg yolk powder, 2.5% cholesterol, 0.5% bile salts, 5% sucrose, 5% peanut, 5% milk powder, and 2% salt. Both diets used in this study were bought from Aoke Xieli Co., Ltd. (Beijing, China). After the treatment for five months, the body weight and fasting glucose level of each mouse were detected. Then the mice were sacrificed, and liver tissues were collected. All samples were stored at −80 °C for further experiments.

All the experimental procedures were approved by the Institutional Animal Care and Use Committee of Animal Center, Institute of Process Engineering, Chinese Academy of Sciences (Beijing, China) and in accordance with the guidelines of the National Act on Use of Experimental Animals (China).

### 4.5. Intraperitoneal Glucose Tolerance Test (IGTT)

An IGTT was conducted after the COS and HFD treatment for five months. Before the test, mice of each group (*n* = 5) were fasted for 12 h and then given an intraperitoneal (i.p.) injection of glucose at a dose of 500 mg/kg body weight. Blood was collected from the tail vein at 0, 15, 30, 60, and 120 min, and the glucose levels were determined using a blood glucometer (Roche Diagnostics, Basel, Switzerland).

### 4.6. Oil Red O Staining

Liver tissues of each group (*n* = 5) were fixed overnight in 4% paraformaldehyde, embedded in paraffin, and made into 4 μm sections. After the deparaffinization and rehydration, the sections were stained with haematoxylin–eosin (HE) for 1 min and then with 0.5% oil red O solution for 30 min at room temperature. The visualized red oil droplets staining in the slides were observed using a Leica DMI4000 B light microscope (Wetzlar, Germany).

For HepG2 cells staining, the cells were treated as before, washed with PBS, and fixed with 4% paraformaldehyde for 60 min. Then, cells were stained with 0.5% oil red O solution and photographed as mentioned above. The experiments were repeated 3 times independently, and 3 replicates were involved in each sample.

### 4.7. RNA Extraction, cDNA Synthesis, and Quantitative Real-Time (qRT)-PCR

Total RNA was extracted from HepG2 cells and liver tissues using TRIzol reagent (Invitrogen, Carlsbad, CA, USA) following the manufacturer’s instructions. Isolated RNA (1 µg) was reverse transcribed into cDNA using a HifiScript cDNA synthesis kit (Takara Bio Inc., Otsu, Shiga, Japan). For qRT-PCR, the reaction was performed using a 7500 Fast Real-Time PCR System (Applied Biosystems, Foster City, CA, USA) with the thermal cycle condition as follows: 95 °C for 2 min; 40 cycles of amplification (95 °C for 15 s, 60 °C for 60 s, 72 °C for 1 min). The experiments were repeated 3 times independently, and 3 replicates were involved in each sample in vitro. Five replicates were involved in each group in vivo. The primer sequences used in this study are listed in Table 1, and β-actin was used as reference gene for calculation of the relative target gene expression using the 2^−ΔΔCT^ method.

### 4.8. Western Blot Analysis

HepG2 cells and liver tissues were homogenized using radio-immunoprecipitation assay (RIPA) buffer (Cell Signaling, Danvers, MA, USA) supplemented with protease inhibitor cocktail (Merck, Darmstadt, Germany). The sample homogenate was centrifuged at 15,000× *g* for 15 min at 4 °C, and the supernatant was collected. The protein concentration was determined using a bicinchoninic acid (BCA protein assay kit (Beyotime, Shanghai, China). The lysate samples (40 µg/lane) were separated by sodium dodecyl sulfate polyacrylamide gel electrophoresis (SDS-PAGE) and transferred to a polyvinylidene fluoride (PVDF) membrane. The membranes were blocked with 5% fat-free milk in Tris-buffered saline with 0.1% Tween-20 (TBST) buffer (10 mM Tris, 150 mM NaCl, 0.1% Tween 20, pH 7.6) for 1 h at room temperature and then incubated with primary antibodies against PPARγ overnight at 4 °C. Next, the membranes were incubated with HRP-conjugated secondary antibodies for 1 h. The protein bands were captured by enhanced chemiluminescence (ECL) (Cell Signaling Technology, Beverly, MA, USA), and the densitometry analysis was performed using an Image J2x software (National Institute of Health, Bethesda, MD, USA). The protein level of PPARγ of each sample was measured 3 times independently in vitro, and 5 samples expressing PPARγ protein of each group in vivo were assessed.

### 4.9. Statistics

Statistical analysis was carried out using SPSS 10.0 package (SPSS Inc, Chicago, IL, USA). Data are presented as means ± SD. Differences between groups were assessed with one-way analysis variance (ANOVA), along with the Tukey–Kramer test. *p* < 0.05 was regarded as statistically significant.

## Figures and Tables

**Table 1:** List of the primer sequences used in RT-PCR analysis.

Gene	Forward Primer (5′-3′)	Reverse Primer (5′-3′)
β-Actin	AGGTGACAGCATTGCTTCTG	GCTGCCTCAACACCTCAAC
IL-6	GGCACTGGCAGAAAACAACC	GCAAGTCTCCTCATTGAATCC
MCP-1	GGGATCATCTTGCTGGTGAA	AGGTCCCTGTCATGCTTCTG
TNF-α	AGGGTCTGGGCCATAGAACT	CCACCACGCTCTTCTGTCTAC
PCK1	CTGCATAACGGTCTGGACTTC	CAGCAACTGCCCGTACTCC
SCD-1	ATACCACCACCACCACCATT	CATACAGGGCTCCCAAGTGT
ACC1	CTGCCATCCCATGTGCTAAT	AGCAGTCGTTCCCCTTCATT
PPARγ	TCGCTGATGCCTGCCTATG	GGAGCACCTTGGCGAACA

## Abbreviations

NAFLDs	nonalcoholic fatty liver diseases
COS	chitosan oligosaccharides
PA	palmitic acid
HFD	high-fat diet
CD	chow diet
IL-6	interleukin-6
MCP-1	monocyte chemoattractant protein 1
TNF-α	tumor necrosis factor-alpha
PCK1	phosphoenolpyruvate carboxykinase-1
SCD-1	stearoyl-CoA desaturase
ACC1	acetyl-CoA carboxylase
